# Safety of Exposure to 0.2 T and 4 Hz Rotating Magnetic Field: A Ten-Month Study on C57BL/6 Mice

**DOI:** 10.3390/cimb46070382

**Published:** 2024-06-26

**Authors:** Hua Yang, Yu Han, Cai Zhou, Shenglan Nie, Mengqing Li, Qinyao Yu, Yunpeng Wei, Xiaomei Wang

**Affiliations:** 1 Department of Physiology, School of Basic Medical Sciences, Shenzhen University Medical School, Shenzhen University, Shenzhen 518055, China; 2 International Cancer Center, Shenzhen University Medical School, Shenzhen University, Shenzhen 518055, China; 3 School of Pharmacy, Shenzhen University Medical School, Shenzhen University, Shenzhen 518055, China

**Keywords:** rotating magnetic field, mice, chronic toxicity, skeletal system, blood, histomorphological examination

## Abstract

Amidst the burgeoning interest in rotating magnetic fields (RMF) within biological research, there remains a notable gap in the scientific evidence concerning the long-term safety of RMF. Thus, this study aimed to investigate the safety of protracted exposure to a 0.2 T, 4 Hz RMF over 10 months in mice. Two-month-old female C57BL/6 mice were randomly allocated to either the RMF group (exposed to 0.2 T, 4 Hz real RMF) or the SHAM group (exposed to 0 T, 4 Hz sham RMF). Throughout the experiment, the murine weekly body weights were recorded, and their behavioral traits were assessed via open field tests. In the final month, a comprehensive evaluation of the murine overall health was conducted, encompassing analyses of blood parameters, histomorphological examination of major organs, and skeletal assessments using X-ray and micro-CT imaging. The murine immune system and lipid metabolism were evaluated through immunochip analysis and metabolomics. Notably, no discernible adverse effects with RMF exposure were observed. Murine body weight, locomotor behavior, organ histomorphology, and skeletal health remained unaffected by RMF. Blood analysis revealed subtle changes in hormone and lipid levels between the SHAM and RMF groups, yet these differences did not reach statistical significance. Moreover, RMF led to elevated serum interleukin-28 (IL-28) levels, albeit within the normal range, and modest alterations in serum lipid metabolites. Conclusively, mice exposed to the 0.2 T, 4 Hz RMF for 10 months displayed no significant signs of chronic toxicity, indicating its potential clinical application as a physical therapy.

## 1. Introduction

The magnetic field (MF) is an essential physical field that is critical to the development and well-being of life on Earth and the formation of contemporary humanity [1]. MF is classified into two categories: static magnetic fields (SMF) and variable magnetic fields (VMF). An SMF is identified by a magnetic field that preserves a consistent intensity and orientation. VMFs display dynamic variations in both intensity and orientation over time, encompassing a rotating magnetic field (RMF), an alternating magnetic field (AMF), a pulsing magnetic field (PMF), and an irregularly variable magnetic field [2]. An RMF is defined as a magnetic field that periodically reverses direction at a constant angular velocity [3].

The clinical use of RMFs has been employed to mitigate bone and joint diseases [4], as they are effective in alleviating osteoporosis [5], improving bone density [6], facilitating bone reconstruction [7,8], and exerting anti-inflammatory and anti-aging effects [9]. A recent study claims that RMFs effectively improve experimental autoimmune encephalomyelitis (EAE), and the plausible mechanism is that an RMF reduces the level of inflammation in EAE mice via the regulation of Treg cell differentiation [10]. Basic studies targeting RMFs are still in progress. Clinical MFs, as a means of disease prevention and treatment, still require prolonged application. However, the scientific foundation for safety investigations on RMF, especially in terms of long-term exposure, is insufficient.

A recent study assessed the acute toxicity of a 16 T SMF in mice, and the results indicated that except for a noticeable increase in MCV and TP in murine blood and transient behavior of tightly circling, no other anomalies were observed in exposed mice [11]. In 2016, B.A. Katsnelson et al. conducted a study that investigated the toxicological effects of sub-chronic exposure to SMFs on rats, as well as the potential combined effects of SMF exposure and fluoride. The rats exhibited mild toxic reactions after sub-chronic exposure to SMFs at 25 mT, and the skeletal system was the primary site of impact. The interaction between SMFs and fluoride in terms of toxicity is a complicated phenomenon, including several mechanisms, such as the single-factor effect and an additive unidirectional effect, along with synergistic effects [12]. Izumi Nishimura et al. investigated the acute and sub-chronic toxicity of 0.1 mT MFs (20 kHz and 60 kHz) in rats, and the results showed that there were moderate alterations in several markers during acute exposure. However, these initial findings were not substantiated in further trials. Besides, no significant differences were observed in any of the indicators between the mice exposed to sub-chronic conditions and the control group. The study demonstrated that both acute and sub-chronic exposure to medium-frequency MFs did not result in any detrimental effects [13]. A further set of studies examined the effects of MF exposure (60 Hz; 0 gausses, 2 gausses, 10 gausses) on hematological and clinical chemical parameters, pineal hormone concentrations, and histopathology in F344/N rats and B6C3F1 mice in an 8-week study [14]. In addition, another study has been performed to investigate the potential teratogenic and reproductive effects of MF on Sprague Dawley (SD) rats. However, none of the aforementioned studies revealed any evidence of detrimental impacts resulting from MFs; only a modest drop in serum melatonin levels in MF-exposed rats was observed, but these changes were deemed to lack biological significance [15].

The intensity of MFs used in both research and therapeutic has undergone a progressive increase with advancements in the understanding of the biological effects of MFs. Furthermore, the range of MFs utilized in previous research and clinical treatment has become more diverse. Our research team successfully constructed an RMF apparatus with an intensity of 0.2 T and a frequency of 4 Hz [10,,16]. Comparable RMFs have been used by numerous researchers for scientific investigation and therapeutic treatment [17,18,19,20]. However, the majority of existing studies on the adverse impacts of MFs have been concentrated on the acute toxicity of intensity below 0.1 T, while there is a notable dearth of scientific evidence on the chronic toxicity of low-frequency RMFs. Thus, this study was carried out to assess the safety of long-term exposure to 0.2 T, 4 Hz RMF in mice.

## 2. Materials and Methods

### 2.1. RMF Characterization

**Figure 1:**
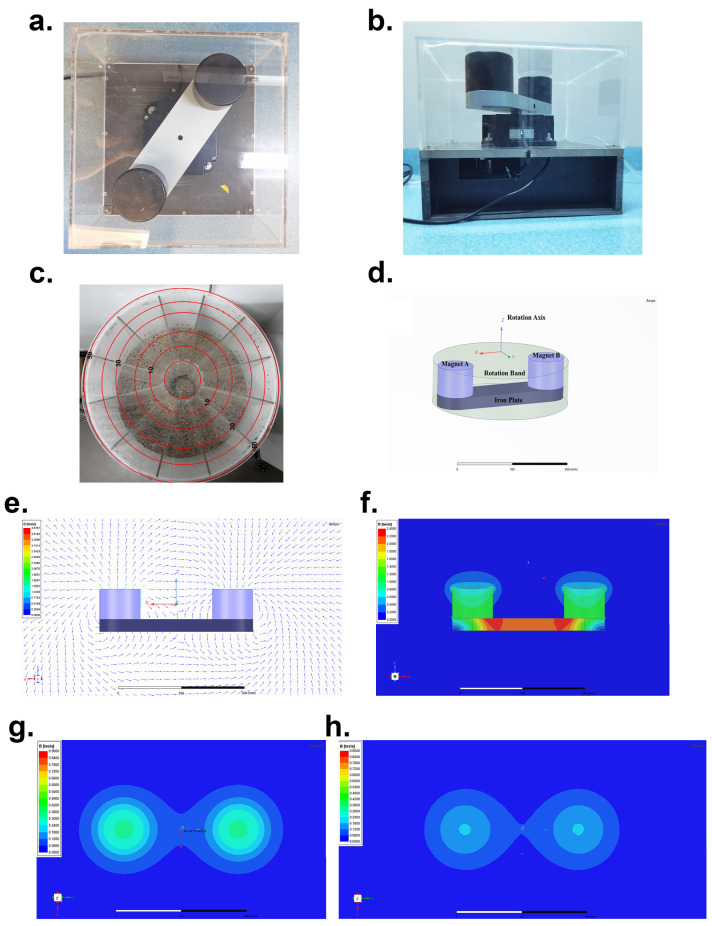
Characterization of the RMF device. (**a**,**b**) The general view of the RMF device (vertical view and lateral view). (**c**) RMF equipment in the animal house. (**d**) The simulation of the core structure of the RMF apparatus. (**e**) The direction of the motion of the magnetic induction lines. (**f**) Distribution of the magnetic induction intensity. (**g**) Simulation of magnetic field strength in the dorsal plane of mice. (**h**) Simulation of magnetic field strength in the footprint plane of mice.

The RMF exposure device used in this study has been previously reported in detail [10]. Briefly, the RMF device comprises two parallel cylindrical neodymium iron boron (NdFeB) permanent magnets with opposite magnetization directions (each with a diameter of 98 mm, a height of 72 mm, and a center-to-center distance of 270 mm, designed by Professor Zhang Xiaoyun of Shenzhen University, Shenzhen, China) (Figure 1a,b,d). The customized individually ventilated cages (IVCs) for the RMF are positioned at a distance of 30 mm from the upper surface of the magnet (Figure 1c). The mice were placed in a treatment container and exposed to an RMF. The ANSYS Maxwell 2020R1 software was employed to simulate the direction of the distribution of magnetic induction lines and the longitudinal magnetic field intensity (Figure 1e,f), as well as the magnetic field intensity at murine podalic flat and murine dorsal flat, respectively (Figure 1g,h).

### 2.2. Materials

Four percent paraformaldehyde was purchased from Biosharp (Beijing, China). Decalcifying fluid with EDTA was acquired from Solarbio (Beijing, China). Safranin-O/Fast green Stain kit, Eosin, and Hematoxylin were obtained from Solarbio (Beijing, China). Isoflurane was acquired from RWD (Shenzhen, China). Diluent for Hematology Analyzer was purchased from Mindray (Shenzhen, China). Mouse Cytokine Quantitative Antibody Microarray (IL-28, IL-1β, IL-2, IL-5, IL-6, IL-10, IL-12p70, IL-13, IL-17, IL-17F, IL-21, IL-22, IL-23, IFN-γ, MIP-3a, TGF-β1, TNF-α, IL-4) was obtained from Raybiotech (Peachtree Corners, GA, USA).

### 2.3. Animals

Twenty-four female C57BL/6 mice (2 weeks old) were purchased from the Guangdong Experimental Animal Center (Guangzhou, Guangdong, China) [SPF, SCXK(G)2018-0002] and raised in an environment with a temperature of 24 ± 2 °C, a humidity level of 60 ± 10%, and a light/dark cycle of 12 h. The mice were randomly divided into two groups: the Sham RMF group (SHAM) and the real RMF group (RMF), with 12 mice in each group. The mice were placed in individual cages and allowed to access food and water freely.

Animal experiments were conducted according to the applicable ethical principles of the Animal Ethical and Welfare Committee (AEWC) of Shenzhen University and the Ministry of Science and Technology of China, as well as the ethical rules outlined in the Guide for the Care and Use of Laboratory Animals (National Institutes of Health, Bethesda, MD, USA). During this study, every effort was made to reduce the number of animals utilized and to minimize animal suffering.

### 2.4. RMF Treatments

In this study, the mice were divided into the RMF group and the SHAM group. In order to exclude the interference caused by non-magnetic factors such as mechanical vibration, two distinct RMF devices were employed, including a 0.2 T, 4 Hz real RMF device and a 0 T, 4 Hz sham RMF device, which exhibit identical appearances and do not interfere with each other. The mice in the RMF group were exposed to the 0.2 T, 4 Hz RMF for 2 h every day, while the mice in the sham group were exposed to the sham RMF (0 T, 4 Hz) for 2 h every day at the same time, all the exposure was sustained over a period of 10 months.

### 2.5. Open Field Test

Open field tests were conducted once a month. The locomotor behavior of mice was videotaped using Ethovision XT software (Noldus, Wageningen, Netherlands, https://www.noldus.com/ethovision-xt (accessed on 20 June 2024)) for 30 min to perform the open field test analysis and evaluate the locomotor activity. Meanwhile, the moving trail of mice was recorded, and the mean velocity of movement was measured.

### 2.6. Blood Routine Examination and Blood Biochemistry

At the end of the RMF exposure session, all mice were anesthetized with isoflurane. Blood samples were then collected from the heart and transferred into tubes containing the anticoagulant EDTA-2K. An automatic blood analyzer (SF-3000; Sysmex Co., Ono, Hyogo, Japan) was employed to detect blood routine indexes. Another automatic analyzer (BS220; Mindray Medical International, China) was applied to detect blood biochemical indicators.

### 2.7. Organ Coefficient Analysis

The mice were anesthetized with isoflurane (RWD, Shenzhen, China) and sacrificed by cardiac puncture, and the heart was then perfused with 0.9% NaCl solution. Organs such as the liver, spleen, kidney, brain, heart, lungs, and ovaries are then collected. Absorb water from the surface of the organ with absorbent paper. The organ coefficient is determined as the ratio of organ weight to total body weight.

### 2.8. Histomorphological Analysis

Three mice from each group were randomly selected for histomorphological analysis. The heart, liver, spleen, lung, kidney, and ovary specimens were collected and fixed in 4% paraformaldehyde for 3 days. These organs were then embedded in paraffin wax and sliced into sections with a sickness of 5 µm using a semi-automatic rotary slicer (RM2245, Leica Biosystems, Heilbrugger, Switzerland). The morphological alterations of the tissues were detected by hematoxylin and eosin (H&E) staining. The images were acquired using a light microscope (BX51, Olympus, Tokyo, Japan).

### 2.9. X-ray Imaging

The mice were subjected to anesthesia with a gas combination of isoflurane and oxygen in a small animal imager (IVIS Lumina XR, Perkinelmer, Waltham, MA, USA). Once it was confirmed that the mice were in a stable anesthetic state, a scan was performed to obtain the bone microstructure imaging and bone mineral density (BMD) of the mice.

### 2.10. Micro-CT Imaging

Microcomputer tomography (micro-CT) imaging of the murine spine was performed on 3 mice from each group. At the ages of 4, 7, and 10 months, three mice in each group were euthanized with isoflurane, and the spine was separated from the cervical spine to the lumbar spine. The tissues were fixed with 4% paraformaldehyde and scanned at an isotropic resolution of 9 μm in a micro-CT imaging apparatus (Quantum GX2, PerkinElmer, Waltham, MA, USA). After scanning, three-dimensional (3D) structure reconstruction of murine tissues was performed by Analyze 14.0 software (Overland Park, KS, USA).

### 2.11. Safranin -O and Fast Green Staining

After RMF exposure for 10 months, the knee joints were extracted after the mice were euthanized with isoflurane and processed into paraffin sections. The knee joint paraffin sections underwent staining with a kit (Solarbio, Beijing, China). Briefly, the nuclei were first subjected to hematoxylin staining (ORIGENE, Beijing, China) solution for 5 min; HCL-ETOH (1%, *v*/*v*) and ammonia-H2O (0.2% *v*/*v*) were applied to separate colors, and the sections were washed in tap water for 10 min. Subsequently, the tissue slices underwent staining with safranin O (2%, *w*/*v*) and fast green solution (0.05%, *w*/*v*). After DDW rinsing, the slides were dehydrated in 95% ethyl alcohol and absolute ethyl alcohol, respectively. Resinous mounting material (BASO, Zhuhai, Guangdong, China) was employed to mount the slides. A light microscope was utilized to obtain the images (BX51, Olympus, Tokyo, Japan).

### 2.12. Immunochip

After RMF treatment for 10 months, the murine serum specimens were collected. After the collection of murine blood, the serum was isolated by a centrifugation process at 4 °C, 3000 r/min for 15 min, and the supernatant was meticulously transferred to a fresh centrifuge tube and preserved at −80 °C. The serum specimens were then subjected to RayPlexTM Bead Arrays Quantitative Multiplex Bead Immunoassay analysis (Abair Technologies Ltd., Shenzhen, China) to detect cytokines and soluble cytokine receptor proteins in serum samples. A volume of 100 μL of serum sample was pipetted into each well, incubated with the detection antibody mixture, and washed. The samples then underwent incubation and washing with Cy3-equivalent labeled streptavidin-amyrin. Finally, the signals were obtained and analyzed using a microarray scanner at Cy3 wavelengths and background-free normalized wavelengths.

### 2.13. Lipid Metabolomics Analysis

After 10 months of RMF treatment, the serum samples obtained from mice were submitted to MetWare Co., Ltd. (Wuhan, China). A comprehensive analysis was conducted on a total of 88 lipid metabolites. The serum specimen was detected using the tandem mass spectrometry (MS/MS, Applied Biosystems QTRAP, Waltham, MA, USA) system and the ultra-performance liquid chromatography (UPLC, Shimpack UFL SHIMADZU CBM30A, Kyoto, Japan). The resultant matrix was implemented concurrently with PLS-DA and OPLS-DA analyses to support profile visualization and differentiation for the respective datasets. The selection of significant variables was based on a conventional criterion: *p* < 0.05.

### 2.14. Statistical Analysis

The results in this study were presented as means and standard deviation (means ± SD). Differences between the two independent group samples were analyzed using the *T*-test. For the indicators that have been dynamically monitored with the extension of study time, a two-way analysis of variance (ANOVA) method has been employed. All the indicators detected underwent a normality test (Shapiro-Wilk test) and the ANOVA to confirm whether the data were normally distributed and the variances were equal. For all statistical tests, *p* < 0.05 was considered to be statistically different. The data plot and statistical analyses were carried out using GraphPad Prism statistical Software (Version 8, GraphPad Software, Inc., Boston, MA, USA).

## 3. Results

### 3.1. Effects of 10-Month RMF Exposure on Clinical Observations, Body Weight, and Behavior in Mice

**Figure 2:**
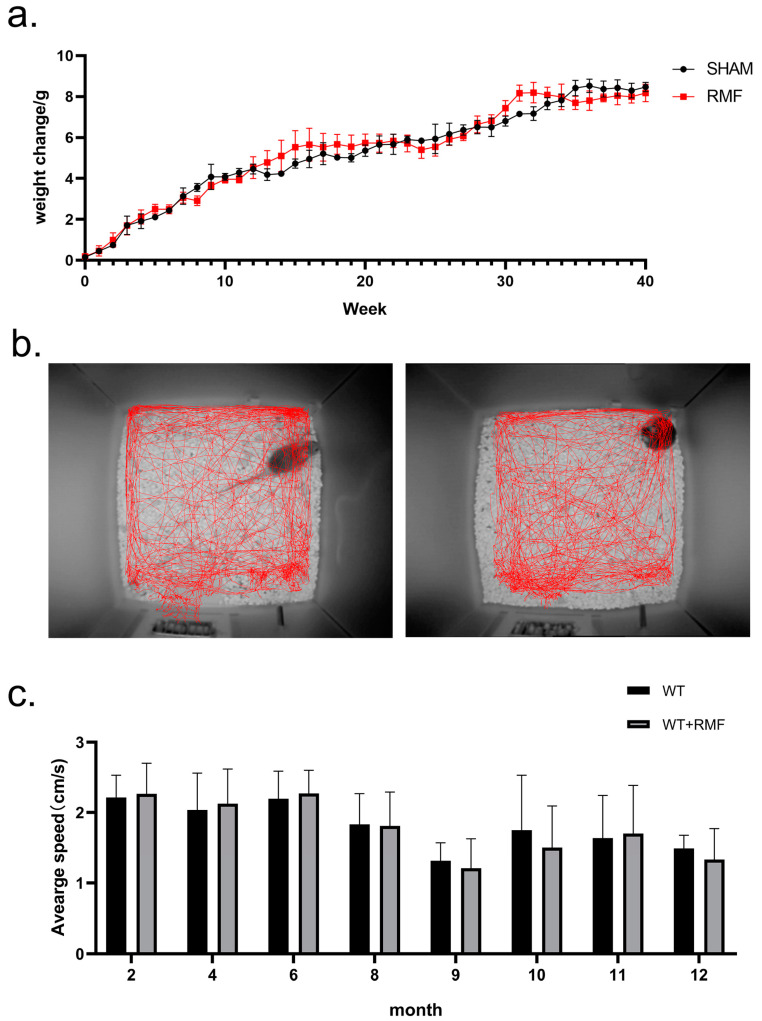
Effects of RMF exposure on body weight and behavior in mice. (**a**) Weekly body weight change of mice in SHAM and RMF groups for 40 weeks (n = 6). (**b**) The locomotion trajectory of mice in open field tests (n = 6). (**c**) The average speed of mice in the open field test was examined at 0, 2, 4, 6, 7, 8, 9, and 10 months, respectively (n = 6).

The body weight results revealed that RMF and SHAM groups experienced comparable changes over the course of the 10-month treatment (Figure 2a). The results of open field tests showed that locomotion trajectory (Figure 2b) and the average locomotion velocity (Figure 2c) in both groups exhibited no significant difference either.

### 3.2. Effects of 10-Month RMF Exposure on Blood Routine Indexes in Mice

**Figure 3:**
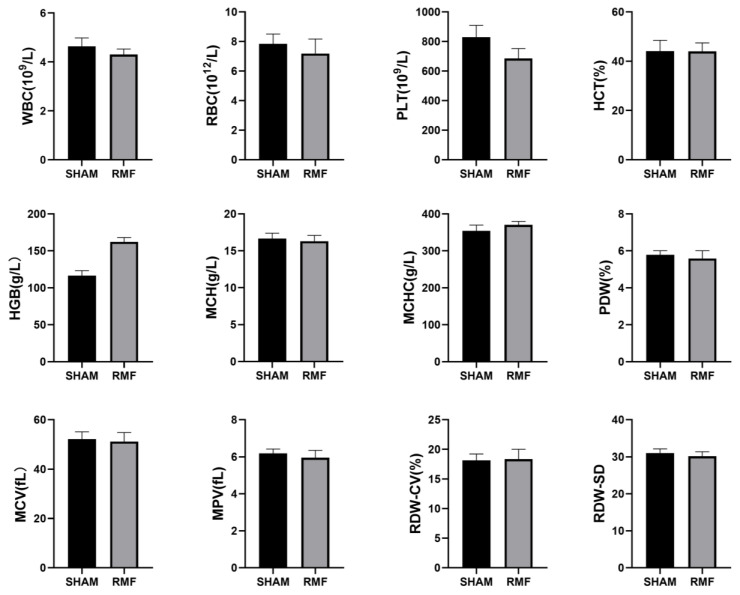
Effects of 10-month RMF exposure on blood routine indices in mice. WBC = white blood cell count; RBC = red blood cell count; PLT = platelet count; HCT = hematocrit; HGB = Hemoglobin; MCH = mean corpuscular hemoglobin; MCHC = mean cell hemoglobin concentration; PDW = platelet distribution width; MCV = mean corpuscular volume; MPV = mean platelet volume; RDW-CV = red blood cell distribution width-coefficient of variation; RDW-SD = red blood cell distribution width standard deviation. (n = 6).

This study aimed to assess the impact of a 10-month exposure to an RMF on mice by measuring various hematological parameters, including white blood cell count (WBC), red blood cell count (RBC), platelet count (PLT), hematocrit (HCT), Hemoglobin (HGB*), mean corpuscular hemoglobin (MCH), mean cell hemoglobin concentration (MCHC), platelet distribution width (PDW), mean corpuscular volume (MCV), mean platelet volume (MPV), red blood cell distribution width-coefficient of variation (RDW-CV), and red blood cell distribution width standard deviation (RDW-SD). The blood routine examination results showed that only the serum hemoglobin (HGB) level in the RMF group exhibited a significant rise when compared to the SHAM group. There were no other significant differences in the blood routine indexes between the SHAM group and the RMF group (Figure 3).

### 3.3. Effects of 10-Month RMF Exposure on Blood Biochemistry Indexes in Mice

Compared to the SHAM group, the serum creatine kinase (CK) and total cholesterol (TC) levels were significantly decreased in the RMF group (Table 1). Except for CK and TC, the other 22 blood biochemical indices had no significant differences between SHAM and RMF groups.

### 3.4. Effect of 10-Month RMF Exposure on Organ Histomorphological Alterations and Organ Coefficients in Mice

**Figure 4:**
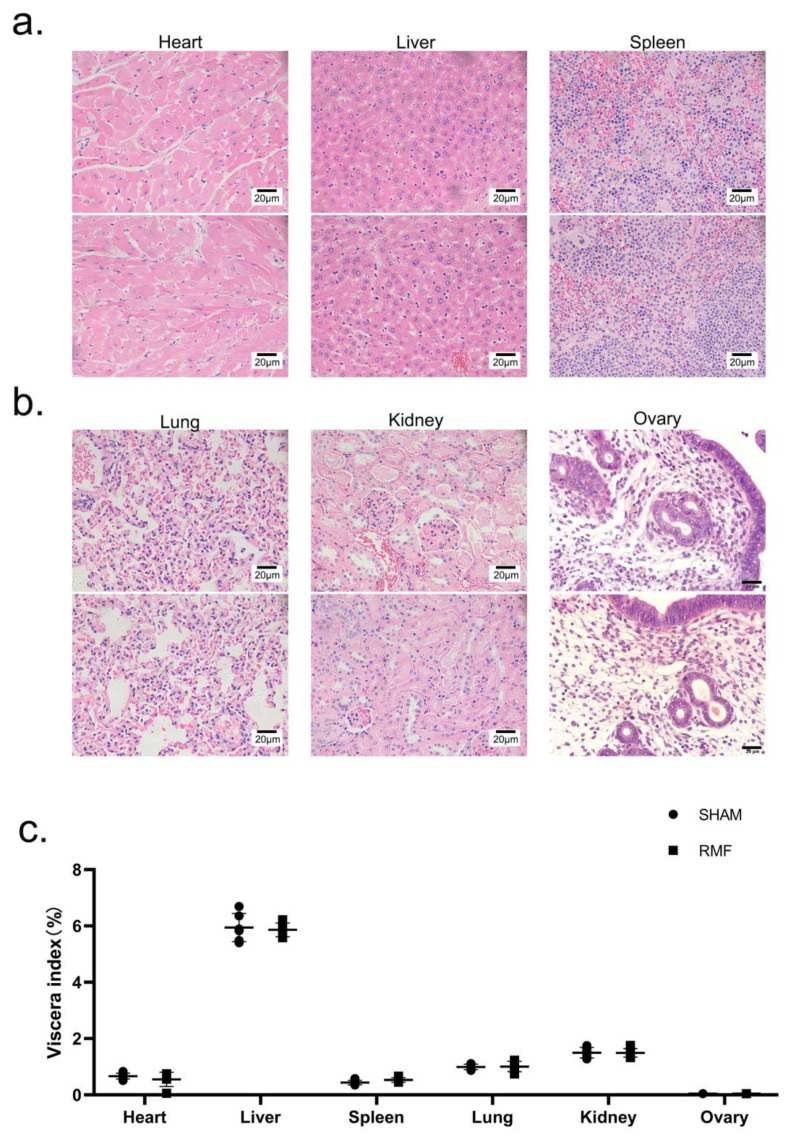
Effect of 10-month RMF exposure on organ histomorphological alterations and organ coefficients in mice. (**a**) The histomorphology of the heart, liver, and spleen stained by H&E staining (scale bar = 20 μm, n = 6). (**b**) The histomorphology of lung, kidney, and ovary stained by H&E staining (scale bar = 20 μm, n = 6). (**c**) The organ coefficients (n = 6).

In necropsy operations, no discernible aberrant pathological alterations were identified in the tissues of murine major organs. Furthermore, the HE staining showed that the histological structure of major organs remained intact, exhibiting well-defined nucleocytoplasmic boundaries, and none of the mice had any evident clinical abnormalities (Figure 4a,b). In addition, the organ coefficients exhibited no statistical differences between the SHAM and RMF groups (Figure 4c).

### 3.5. Effects of 10-Month RMF Exposure on the Skeletal System in Mice

**Figure 5:**
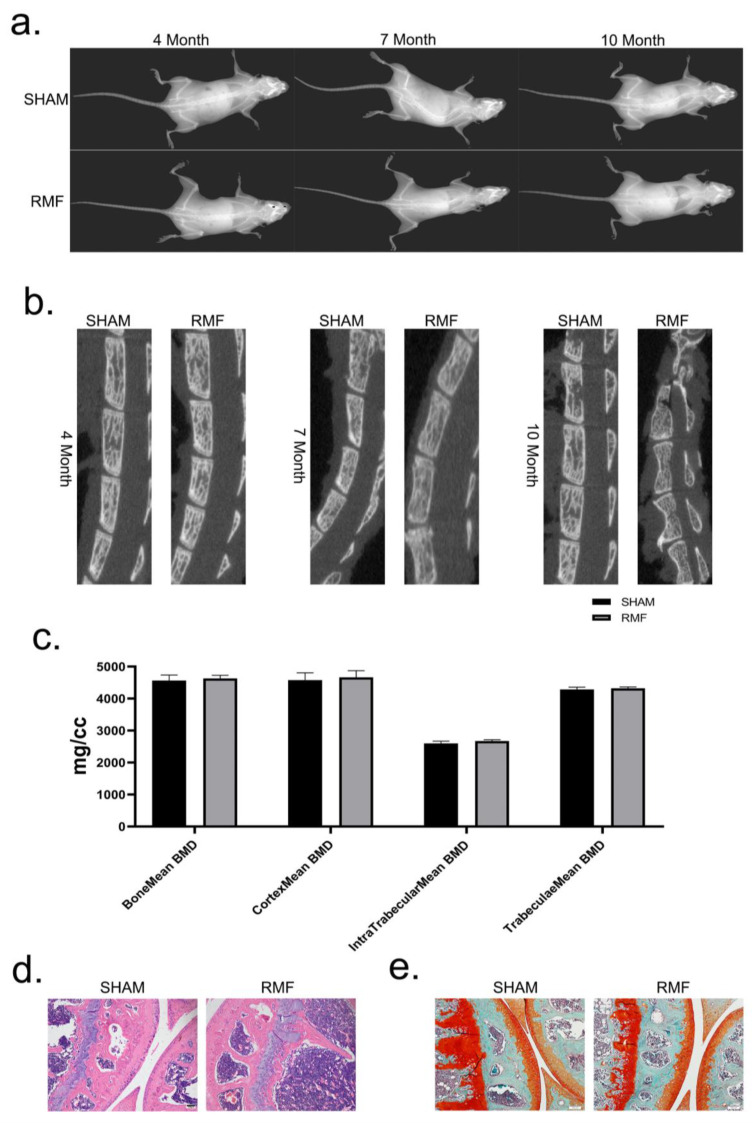
Effects of RMF exposure on the skeletal system in mice. (**a**) X-ray images of mice from the two groups. (**b**) Micro-CT imaging of the spinal column in mice from the two groups. (**c**) Mean bone mineral density (BMD), including Bonemean BMD, Cortexmean BMD, IntraTrabecularMean BMD, and TrabeculaeMean BMD of the mice from the two groups (n = 6). (**d**) H&E staining of murine knee joints (n = 6, scale bar = 100 μm). (**e**) Safranine O and fast green staining of murine knee joint (n = 6, scale bar = 100 μm).

At the 4th, 7th, and 10th month, comprehensive X-ray imaging of the whole body (Figure 5a) and micro-CT imaging (Figure 5b) of the spine were conducted on both the SHAM group and the RMF group, and the results demonstrated no discernible skeletal pathological alterations. The BoneMean BMD, CortexMean BMD, IntraTrabecularMean BMD, and TrabeculaeMean BMD between the SHAM and RMF groups had no statistical differences (Figure 5c). Subsequently, the knee joints of mice were stained with H&E (Figure 5d) and safranine O/fast green (Figure 5e) staining; the results also showed no evidence of detrimental effects of RMF exposure.

### 3.6. Effects of 10-Month RMF Exposure on the Immune System in Mice

The effects of a 10-month RMF exposure on the immune system of mice were assessed using an immune-chip assay. The results showed that only serum IL-28 levels in the RMF group significantly increased compared to the SHAM group (Table 2). In addition, the other 17 inflammatory cytokine levels showed no significant differences between the SHAM and RMF groups.

**Table 2:** Serum levels of inflammatory cytokines in the SHAM group and the RMF group.

Protein ID	SHAM (pg/mL)	RMF (pg/mL)
IL-28 **	2.79 ± 1.70	15.94 ± 9.73
IL-1β	21.83 ± 3.09	32.57 ± 12.51
IL-2	10.09 ± 0.47	11.91 ± 2.70
IL-5	33.27 ± 20.43	10.02 ± 3.36
IL-6	33.75 ± 6.14	29.12 ± 3.6
IL-10	88.34 ± 12.79	95.65 ± 14.39
IL-12p70	42.96 ± 2.90	45.81 ± 6.53
IL-13	12.25 ± 2.21	10.85 ± 0.98
IL-17	8.92 ± 3.34	12.31 ± 1.98
IL-17F	7.13 ± 0.34	9.14 ± 1.27
IL-21	2.31 ± 0.74	2.62 ± 1.21
IL-22	0.04 ± 0.01	0.05 ± 0.02
IL-23	281.69 ± 55.45	303.54 ± 54.33
IFNg	50.73 ± 5.29	54.43 ± 6.60
MIP-3a	16.36 ± 5.30	15.49 ± 7.36
TGF-β1	502.92 ± 34.42	744.83 ± 166.80
TNF-α	7.22 ± 1.63	5.98 ± 1.63
IL-4	0.88 ± 0.14	0.85 ± 0.07

Immunochip technology provided by RayBiotech, Inc. (Guangzhou, China). n = 6, ** *p* < 0.01.

### 3.7. Effect of 10-Month RMF Exposure on Lipid Metabolism in Mice

**Figure 6:**
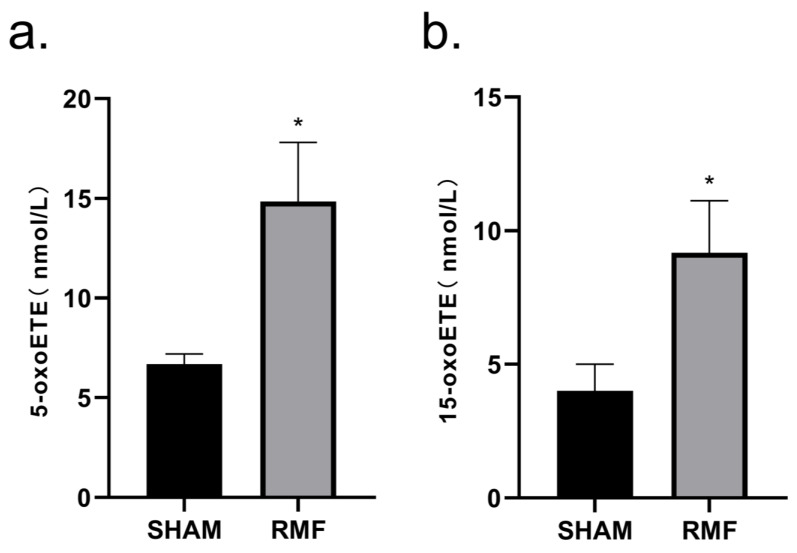
Effect of 10-month RMF exposure on lipid metabolism in mice. (**a**) Serum 5-oxoETE levels in the mice from the SHAM group and the RMF group at the 10th month. (**b**) Serum 15-oxoETE levels in the mice from the SHAM group and the RMF group at the 10th month. n = 6, * *p* < 0.05.

Lipid metabolomics analysis was utilized to assess the effects of 10-month RMF exposure on lipid metabolism in mice. A total of 88 serum lipid metabolite indicators were examined, among which serum 5-oxoETE (Figure 6a) and 15-oxoETE (Figure 6b) levels in the RMF group of mice were higher compared to SHAM mice. Other than that, there were no noticeable differences among the other parameters in the two groups.

## 4. Discussion

The use of RMF is becoming prevalent in scientific studies and clinical treatments. Due to the limited availability of biological studies on the comprehensive risk assessment of long-term RMF exposure, the safety of a 10-month RMF (0.2 T, 4 Hz) exposure was investigated in mice in this study. During the 10-month experiment, mice in the RMF and SHAM groups exhibited almost identical patterns in body weight trends. Furthermore, no significant alterations in the physical characteristics or behavioral patterns of mice were observed between these two experimental groups, indicating inconspicuous chronic toxicity of RMF in mice.

The health states of mice can be reflected by blood-related indices. Compared to the SHAM group, the RMF group displayed a little increase in blood routine HGB index levels and a slight decrease in blood biochemical CK and TC index levels. According to the study of Charles E Wiedmeyer and Olaf Boehm et al., it seems that the HGB, CK, and TC indices exhibit a typical physiological fluctuation range in the RMF and SHAM groups in this study [21,22]. This indicates that 10-month RMF exposure has minimal impact on blood indices, which is consistent with the results of previous studies [23,24,25].

In addition, no statistically significant differences were observed in the absolute organ weights and organ coefficients when comparing the RMF and SHAM groups of mice. In addition, there were no noticeable clinicopathological changes identified in H&E-stained sections of the heart, liver, spleen, lung with bronchi, and kidney in both SHAM and RMF groups. These findings imply that long-term RMF exposure does not induce organic lesions in mice, aligning with the results of toxicity studies using medium and high-frequency MFs [13,24,26,27]. Pathological changes, including tumors, lymphocyte aggregates, and inflammatory hepatic lesions, have been observed in rats after MF exposure, while it is crucial to note that there is insufficient evidence to establish a direct correlation between the pathological alterations and RMF exposure in these studies [28,29].

MFs have important clinical application-value in the treatment of skeletal diseases, including modulating bone metabolism, promoting osteogenesis, facilitating bone damage repair, and enhancing bone density [30,31,32]. Therefore, it is necessary to investigate the potential harmful effects of RMF on the skeletal system. This study showed that long-term RMF did not influence the skeletal system of mice, suggesting that the effects of RMF on the skeletal system of normal mice are minimal, and RMF does not perpetually act unidirectionally on specific functions, such as enhancing bone density and facilitating bone formation. These findings coincide with the studies of other researchers on the toxicity of MF on the skeletal system [12,33].

The impact of RMFs on the immune system-has been extensively investigated, revealing its potential therapeutic effects in different immune-related disease models. RMFs have been reported to modulate the levels of inflammatory cytokines, such as interleukin-1β (IL-1β), tumor necrosis factor-α (TNF-α), IL-6, IL-8, IL-4, and IL-10, and these studies have explored the effects of almost all types of MFs on immune function [34,35,36,37]. However, current studies on the toxic impacts of MFs lack comprehensive data on the effects on the immune system of normal mice. This study explored the effects of RMF on the immune system of normal mice and found no discernible differences in the levels of 17 cytokines detected between the RMF group and the SHAM group, with the exception of elevated IL-28 levels. Interestingly, it is noteworthy that this may be the first report of the alterations in IL-28 levels after RMF treatment. IL-28, like type I interferon, is induced by viral infections and displays antiviral activity [38]. However, it ought to be emphasized that the levels of IL-28 in both groups of mice in this study did not surpass thresholds typically observed in pathological inflammatory states [39,40]. Meanwhile, no additional corroborative-evidence was obtained to prove that an RMF has toxic effects on the immune system of mice.

In recent years, with the increasing research on the effects of MFs on lipid metabolism, the serum lipid metabolites of RMF mice and SHAM mice were also investigated in this study [41,42]. Serum 5-oxoETE and 15-oxoETE levels exhibited an increase after RMF treatment compared to the SHAM group, but with minimal differences between the two groups and large standard deviations within the groups, which displayed no significant difference between the two groups. As both 5-oxoETE and 15-oxoETE are members of the arachidonic acid class, which play essential parts in humoral immunity, the abnormal elevations of these compounds may suggest the presence of infection. However, the levels of these two substances in the RMF group in this particular instance were much lower than those under pathological conditions, demonstrating that an RMF exerted no noticeable adverse effects on serum lipid metabolism in mice.

Although this study has revealed several interesting results, there are certain limitations. Since there are relatively few investigations of low-dose RMF and almost no studies of its long-term toxicity, there is no sufficient research basis. In this work, only the safety of RMF in female C57BL/6 mice was examined. Although female mice are more sensitive to toxic reactions than males [43,44,45], an in-depth assessment of the safety--of RMF is still required with comparisons between male and female mice. This investigation only explored the safety assessment of mice in RMF treatment at a fixed intensity (0.2 T) and frequency (4 Hz), which can be followed up by setting more magnetic field intensities and frequencies to be performed. In this work, only the effects of RMF on the murine weight and behavioral alterations were dynamically monitored. In the future, the effects of RMF on indicators, including cytokines and biochemical indices, in mice should be further dynamically evaluated.

This work has conducted a 10-month safety study of 0.2 T and 4 Hz rotating magnetic field (RMF) on C57BL/6 mice. This study has certain clinical and practical significance: RMFs, as a non-invasive physical therapy modality, are safe and convenient. The relative convenience of patient cooperation in the clinical application of RMFs facilitates the implementation of long-term treatment. The primary measurements to guide future research, the outcomes that ought to be considered, and the appropriate type of study for each outcome are presented in the following table (Table 3):

The primary outcome measurement guiding future research associated with RMFs is the one that best reflects the effect of the intervention or the phenomenon being investigated, including biological effects and safety assessments. The intensity, frequency, and duration of RMF exposure, and its sensitivity and relevance to the disease, ought to be considered to select the appropriate type of study to assess the impact of RMF. Consideration should also take into account the gender, age, genetic background of the subjects, and other variables that have the potential to influence the results. In the design of future studies, the above-mentioned measurement ought to be taken into consideration, and the appropriate type of study ought to be selected according to the purpose and the specific conditions of the study.

In this study, C57BL/6 mice under long-term RMF (0.2 T, 0.4 Hz) exposure did not exhibit significant toxic impairment. However, the subsequent research and application of RMF still have a certain developmental space. Although the preparation process of RMF devices is relatively mature, the mass production and the realization of clinical translation still require further efforts. In the following research, the programmed and personalized treatment plans can be designed based on different diseases and stage classifications. Meanwhile, different magnetic field intensities and frequencies are customized to achieve precise treatment with RMFs. The effect of RMFs on the organism is complex, and it probably exerts multiple regulatory effects on the internal environment of cells and tissues. Therefore, more in-depth investigations are desired to understand the exact mechanism of the effects of RMF on the organism. In the subsequent studies, the effects of RMF exposure on different cell lines in vitro can be observed, and the diseases for which RMFs have a palliative effect can be explored in depth. In general, RMF, as a non-invasive new type of physiotherapy, can be employed in conjunction with immunotherapy, drug therapy, surgical therapy, photothermal therapy, and other therapeutic methods to achieve more effective treatment of diseases. To better achieve clinical translation of RMFs, further optimization of systems such as therapeutic systems, therapeutic devices, standardized practices, and safety assessments is a necessity.

## 5. Conclusions

This study provides a plethora of findings on the safety of RMF long-term exposure in mice. The results indicated that long-term RMF exposure had minimal effects on hematological and hematobiochemical parameters in normal mice and caused a modest increase in serum IL-28, 5-oxoETE, and 15-oxoETE levels. However, the values of these parameters altered by RMFs remained within the normal reference interval. Other than that, RMF had no further chronic adverse effects. In summary, there is insufficient evidence to support any hypothesis suggesting the toxicity of long-term RMF exposure in mice, and RMF may have the potential to be used as a relatively safe approach to scientific research and clinical treatments.

## Figures and Tables

**Table 1:** Effects of 10-month RMF exposure on blood biochemistry indices in mice.

Indices	SHAM	RMF
AST (U/L)	150.15 ± 52.66	149.85 ± 40.28
ALB (g/L)	26.85 ± 1.30	23.1 ± 3.66
ALT (U/L)	23.375 ± 6.09	22.8 ± 5.25
GLU (mmol/L)	6.675 ± 0.14	6.7225 ± 0.98
TG (mmol/L)	2.13 ± 0.41	1.725 ± 0.68
UREA (mmol/L)	6.63 ± 0.99	5.64 ± 2.52
CR (μmol/L)	24.6 ± 5.32	24.6 ± 7.53
UA (μmol/L)	116.825 ± 23.12	112.55 ± 72.00
ALP (U/L)	40.05 ± 24.46	36.325 ± 11.43
LDH (U/L)	559.8 ± 79.52	564.6 ± 89.01
CK (U/L) *	987.25 ± 229.59	648.325 ± 52.42
TC (mmol/L) *	3.525 ± 0.06	2.505 ± 0.61
T-BIL (μmol/L)	4.395 ± 0.81	4.515 ± 1.68
γ-GT (U/L)	1.375 ± 0.75	1.425 ± 0.39
D-BIL (μmol/L)	0.61 ± 0.31	0.88 ± 0.67
α-HBDH (U/L)	351.2 ± 105.08	433.65 ± 65.91
CKMB (U/L)	820.825 ± 386.04	798.325 ± 155.45
AG	0.929 ± 0.01	0.92125 ± 0.03
LDL-C (mmol/L)	0.3115 ± 0.08	0.2855 ± 0.11
HDL-C (mmol/L)	3.56225 ± 0.49	3.48575 ± 0.75
GLB (g/L)	30.855 ± 0.91	29.9775 ± 1.67
TP (g/L)	70.0525 ± 17.00	74.2075 ± 11.81

AST = aspartate transaminase; ALB = albumin; ALT = alanine transaminase; GLU = glucose; TG = triglyceride; UREA = urea; CR = creatinine; UA = Uric Acid; ALP = alkaline phosphatase; LDH = lactate dehydrogenase; CK = creatine kinase; TC = total cholesterol; T-BIL = total bilirubin; γ-GT = γ-glutamine; D-BIL = direct bilirubin; α-HBDH = α-hydroxybutyrate dehydrogenase; CKMB = creatine kinase-MB; AG = anion gap; LDL-C = low-density lipoprotein cholesterol; HDL-C = high-density lipoprotein cholesterol; GLB = globulin; TP = total protein. n = 6. * *p* < 0.05.

**Table 3:** The primary measurements to guide future research.

Primary Outcome Measure	Outcomes	Types of Studies
body weight changes	body weight monitoring	longitudinal study regular measurements and recordings
behavioral traits	open-field experiments	in vivo experiments video tracking analysis software
biological effect	cell proliferation differentiation apoptosis	cell culture CCK8, WB flow cytometry
physiological impact	blood pressure heart rate respiration	in vivo experiments physiological monitoring
blood and biochemical markers	blood components biochemical markers	hematological and biochemical analyses (automated hematology analyzers)
histopathological changes	the effect of RMF on the organization of major organs	H&E staining microscopic observation
skeletal system effects	bone density bone microarchitecture bone metabolism	X-ray, micro-CT bone histomorphometric analysis
immune system evaluation	immune cell activity cytokine levels immune response	immunochip technology flow cytometry
lipid metabolic changes	serum lipid metabolites	mass spectrometry chromatography
dose-response relationship	RMF with different intensities and frequencies	system dose-effect study
safety and risk assessment	the safety and potential risks	experimental data epidemiological studies
statistical analysis	statistical significance and reliability	statistical methods statistical software *t*-test, ANOVA

## Data Availability

The experimental data sets generated and/or analyzed during the current study are available from the corresponding author upon reasonable request.
